# Effect of An Image Resolution Change on the Effective Transport Coefficient of Heterogeneous Materials

**DOI:** 10.3390/ma12223757

**Published:** 2019-11-15

**Authors:** Abimael Rodriguez, Romeli Barbosa, Abraham Rios, Jaime Ortegon, Beatriz Escobar, Beatriz Gayosso, Carlos Couder

**Affiliations:** 1 División de Ciencias e Ingeniería, CONACYT-Universidad de Quintana Roo, Boulevard Bahía s/n, Chetumal, Quintana Roo 77019, Mexico; abima777@gmail.com; 2 División de Ciencias e Ingeniería, Universidad de Quintana Roo, Boulevard Bahía s/n, Chetumal, Quintana Roo 77019, Mexico; jortegon@uqroo.edu.mx; 3 Instituto Politécnico Nacional, Escuela Superior de Ingeniería Mecánica y Eléctrica, Av. Luis Enrique Erro S/N, Unidad Profesional Adolfo López Mateos, Zacatenco, Delegación Gustavo A. Madero, Ciudad de México 07738, Mexico; androidmisraim@gmail.com; 4 CONACYT- Unidad de Energía Renovable, Centro de Investigación Científica de Yucatán, C 43 No 130, Chuburná de Hidalgo, Mérida 97200, Mexico; bem08@hotmail.com; 5 Instituto Politécnico Nacional, Centro de Desarrollo Aeroespacial, Belisario Domínguez 22, Col. Centro, Del. Cuauhtémoc, Ciudad de México 06010, Mexico; betty.gayosso@gmail.com (B.G.); ccouder@hotmail.com (C.C.)

**Keywords:** decimation, effective transport coefficient, simulated annealing, statistical descriptors

## Abstract

Electrochemical electrodes comprise multiple phenomena at different scales. Several works have tried to model such phenomena using statistical techniques. This paper proposes a novel process to work with reduced size images to reconstruct microstructures with the Simulated Annealing method. Later, using the Finite Volume Method, it is verified the effect of the image resolution on the effective transport coefficient (ETC). The method can be applied to synthetic images or images from the Scanning Electron Microscope. The first stage consists of obtaining the image of minimum size, which contains at least 98% of the statistical information of the original image, allowing an equivalent statistical study. The image size reduction was made by applying an iterative decimation over the image using the normalized coarseness to compare the amount of information contained at each step. Representative improvements, especially in processing time, are achieved by reducing the size of the reconstructed microstructures without affecting their statistical behavior. The process ends computing the conduction efficiency from the microstructures. The simulation results, obtained from two kinds of images from different materials, demonstrate the effectivity of the proposed approach. It is important to remark that the controlled decimation allows a reduction of the processor and memory use during the reconstruction and ETC computation of electrodes.

## 1. Introduction

Electrodes are essential in fuel cells to produce electrical energy. The design of electrochemical electrodes requires that models consider multiple phenomena at different scales. This is the case of a proton exchange membrane fuel cell (PEMFC) electrode [1]. In a PEMFC electrode, the reactant gases are transported from the border between catalytic layer (CL) and the so-called gas diffusion layer (GDL) up to the reacting catalytic sites through pores formed between the primary components during fabrication. While the catalyst has the role of promoting the proper electrochemical reactions, the carbon collects and conducts the produced electrons, and the ionomer should conduct protons generated or consumed by the proper reaction. At the nanoscale, a region commonly referred to as a three-phase zone should be studied to stimulate the electrochemical reaction [2]. Nevertheless, at the microscale, the PEMFC electrode can be modeled by agglomerates in a porous matrix [3], in this scale, a Scanning Electron Microscope (SEM) produces high-resolution images of the microscopic structure. Recently, Ortegon et al. presented a classification approach based on Support Vector Machines (SVM) to generate a binarized image from grayscale SEM images of PEMFC electrodes [4]. This heuristic method allows for obtaining a better match with the user’s point of view regarding the solid (agglomerates) and void (porous) phases.

Moreover, considering its structure and composition, a PEMFC electrode is defined as a random heterogeneous material (RHM). This heterogeneity arises from the fact that it is constituted by different phases, being “a phase” an identifiable domain with its own properties that differentiate it from the rest of the other phases in the CL (i.e., voids, other solid material, gases or liquids). Proportionality coefficients for mass, energy, and charge transport in a heterogeneous material are significantly affected by the properties of its phases. For this reason, an effective transport coefficient (ETC) is defined for a heterogeneous material as a proportionality coefficient, which characterizes the domain of the material. For a randomly formed heterogeneous material with *n* phases, a general ETC, $\Gamma_{eff}$ is:

(1) $$\Gamma_{eff}=f\left(\Gamma_{1},\;\Gamma_{2},\;\ldots ,\;\Gamma_{n};\;\phi_{1},\;\phi_{2},\;\ldots ,\;\phi_{n};\;\Omega^{*}\right)$$

where subscript of variables denotes its respective phase, *Γ* is the proportionality constant for that phase, ϕ is the phase volume fraction and $\Omega^{*}$ is the microstructural information of the domain [2]. The review of the literature showed that there are different mathematical relationships to determine ETC’s [5,6,7]. However, a powerful technique to compute ETC is through a microstructural reconstruction, a differential equations model of the transport phenomenon and its numerical solution [8,9,10]. Hence, it can be numerically solved with different methods, such as Finite Differences Method (FDM) or Finite Volume Method (FVM). FVM works with reconstructed volumes, for different scale of synthesized materials and the modeling of transport phenomena in 2D or 3D heterogeneous media [1].

A reconstruction method based on simulated annealing (SA) is used due to its flexibility to take into account different parameters [11,12]. FVM has been tested to represent a volume in order to calculate a highly accurate ETC under different sceneries [5,6,7,8,9,10]. However, the reconstruction of volume and computation of ETC needs vast computational resources. In previous work, a gradual decimation process was developed in order to obtain the image of minimal size without losing statistical information [13]. The method, based on normalized coarseness and the correlation length, indicates the maximum number of steps ($k_{max}$) of a gradual decimation process. This method diminishes the time consumed by characterization, reconstruction, and simulation processes.

The present work presents an extensive and robust method to compute ETC from the electrode’s SEM images. It comprises a progressive and sequential decimation, statistical characterization, reconstruction, and conduction efficiency determination. In order to prove the proposed method, it is analyzed the effect of the image decimation process on the ETC. Binary images, from synthetic and experimental SEM electrodes, have been decimated and studied. The SA reconstruction method was performed to generate an ensemble of ten random configurations of the same sample. Later, FVM is used to determine a conduction efficiency based on the charge transport continuity equation. Details of the conduction efficiency determination, decimation and reconstruction process and statistical behavior of the microstructure are presented. The conduction efficiency average and the error obtained for the ensemble, in both original and decimated microstructures are presented for four case studies. Results show that the proposed method allows reducing the processing time and use of RAM memory without losing relevant statistical microstructure information.

## 2. Methods and Materials

**Figure 1:**
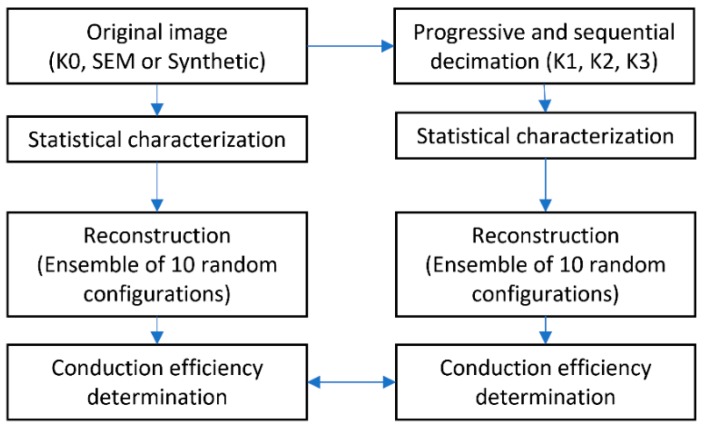
Diagram of the methodology used.

Different techniques have been developed to improve the reconstruction of volumes from SEM micrographs [14]. The main purpose of the methodology used in the present work is to reduce the size of the used SEM image and evaluate its statistical and ETC response. The methodology is shown in Figure 1. Progressive and sequential decimation process is applied to obtain an image of minimal size (in pixels). In this work, random bilinear decimation is implemented [13]. Statistical characterization is obtained with the following correlation functions (descriptors): two-point correlation ($S_{j}$), line-path correlation ($L_{j}$), and pore-size distribution ($P_{j}$). The normalized coarseness was used to evaluate each decimated image, and the correlation length was the key to determine the optimal decimation step. SA reconstruction was applied to obtain one ensemble (*Ω*) that contains ten realizations (*ω*) of each set of material, i.e., the original image or decimated image. Finally, the conduction efficiency ($\varepsilon_{k}$) is determined through FVM. The main outcome of this proposed methodology is the comparison of the $\varepsilon_{k}$, for both: averages of the *Ω* ensembles and variance of the ten *ω* realizations. 

Images from SEM and images digitally generated were binarized, decimated and studied. In both cases, planar materials formed by solid-phase (*j = 1*) and void phase (*j = 0*) are considered. For a statistical study, it is indispensable to define the space and the basic mathematics of the domain considering the characterization, reconstruction, and ETC determination [7].

For a realization *ω* of possible states of a given set of material (statistical reconstruction), it is necessary to take into account the domain of the material v belonging to our measurable space $R^{D}$, of volume *V*, which is partitioned in the random phases *j*. The region $v_{j}\left(\omega \right)$ and its respective volumetric fraction $\phi_{j}\left(\omega \right)$ can be defined. In this work, the studied materials are represented by two-dimensional (2D) binary images, where matrix *A* represents the discretized binary field, containing *N* columns and *M* lines.

The image, with the full resolution, turns out to be the original image of the material, which is represented by the matrix $A_{0}$. From this matrix $A_{0}$, statistical descriptors will be used as reference functions (objective functions) for the realization *ω* in an ensemble *Ω*, of random series (*W*) from the same material, for each *k* decimation step, in this work *W = 10*.

The size reduction strategy has been previously published [13]. In Figure 1, the original image (matrix $A_{0}$, with size $M_{0}\times N_{0}$) has a decimation step *k = 0*, afterward, the image is reduced for its progressive and sequential evaluation from *k = 1* to *k = 4*. In each *k*-th step of the decimation, the resulting matrix $A_{k}$ is statistically characterized. The comparison of the normalized statistical response, between the original image and the decimated one, allows the determination of the deviation of the specific descriptor *β*. On the other hand, the conduction efficiency ($\varepsilon_{k}$) is determined through the ETC, which is numerically solved with FVM. Every *k*-th step of decimation, for each realization ω ∈(1, W), is made to compare the response with respect to the original image and determine the effects of the resolution change. The main numerical methods used are detailed in the following sections.

### 2.1. Statistical Descriptors and Reconstruction

The study of the ETC in RHM benefits from the techniques of micrography and statistical descriptors. There are a wide variety of statistical descriptors, generically referred to as microstructural correlation functions [7]. In this work, $S_{j}$,$\;L_{j}$ and $P_{j}$ correlation functions are used to the statistical characterization, and $S_{j}$ and $L_{j}$ are used in the stochastic reconstruction. These statistical descriptors are fully detailed on [13]. In this paper, it is important to remark the concept of normalized statistical descriptors ($F_{\beta ,j}$) and the average correlation functions of each Ω (ω) ensemble. Equations (2)–(4) define the $F_{\beta ,j}$ in the function of a displacement (or distance) r:

(2) $$F_{1,j}=\frac{{\langle I_{j}\left(x\right)I_{j}\left(x+r\right)\rangle }_{j}^{\left(2\right)}\left(r\right)-\Phi_{j}^{2}}{\Phi_{j}\left(1-\Phi_{j}\right)}$$

(3) $$F_{2,j}=\frac{\left\lfloor \int_{0}^{1}I_{j}\left(x+\alpha r\right)d\alpha \right\rfloor }{\Phi_{j}}$$

(4) $$F_{3,j}=\frac{\Phi_{j}\langle \lfloor \frac{1}{V_{D}}\int_{V_{D}}^{}I_{j}\left(x+\alpha r\right)dV_{D}\rfloor \rangle }{s}$$

where the *β* index is associated with the descriptor, β=1 is taken for the $S_{j}$ function, β=2 for the $L_{j}$ function and β=3 for the $P_{j}$ function, *j* index specifies the phase, $I_{j}$ is the index function, $V_{D}$ and $dV_{D}$ are the volume of a D-dimensional unit sphere and a differential volume, in our local 2D dimensional domain $dV_{D}=\alpha \;d\alpha \;d\theta$, α∈(0,1) and θ∈(0,2π). Changes in statistical information are reported in this paper as the global averages of the $S_{j}$ and $L_{j}$ normalized correlation functions, determined for the *Ω (ω)* ensemble, as described in Equation (5):

(5) $$F_{\left(\Omega ,r\right)}=\frac{1}{20}\overset{10}{\underset{\omega =1}{\sum }}\overset{2}{\underset{\beta =1}{\sum }}F_{\beta ,j}\left(r,w\right)$$

On the other hand, the SA method was applied for the reconstruction of each ω realization. SA generates a system that has the same statistical correlation functions as a specified reference system. In the literature, the details of the mathematical formulation and algorithms are reported [9]. In this work, the evolution of the error (or “energy”) during the SA reconstruction process is presented, for an interval of the SA statistical moments, and it is also compared with the *k*-th decimation step, of all the ensembles of the materials studied. SA error (E_SA_) is defined in Equation (6),

(6) $$E_{\mathrm{SA}}=\overset{}{\underset{r}{\sum }}{\left[F^{\prime }\left(r\right)-F\left(r\right)\right]}^{2}$$

where F(r) is the “reference” and $F^{\prime }\left(r\right)$ is the “current” statistical descriptors of the SA reconstruction method [9]. In this work, $S_{j}$ and $L_{j}$ functions are used, with the same statistical weight, for both phases. The normalized coarseness function, $C_{k}^{*}$, contains the information of the statistical descriptors on the progressive increment of decimated *k*-th process, it is defined in Equation (7). When the *k*-th decimation step is incremented, $C_{k}^{*}$ tends to zero.

(7) $$C_{k}^{*}=\frac{\sigma \left(2^{k}\right)}{\sqrt{\emptyset_{0}\emptyset_{1}}}$$

### 2.2. Decimation Process 

The decimation process consists of the spatial reduction of the binary image. A spatial reduction, refers to the number of pixels in the image, resulting in the loss of information contained in the image. The level of decimation is denoted with *k*, which starts with *k* = 0 (original image) and sequentially incrementing by 1 until reaching the maximum value of *k* considered (*K*). The evolution of *k* is reflected in an image with a reduced number of rows ($M_{k}$) and columns ($N_{k}$) of the original image given by *M_k_* = *M_0_*/*2^k^* and *N_k_* = *N_0_*/*2^k^*. As expected, the image detail decreases as *k* increases. 

In previous work [13], three different decimation strategies (random, bilinear and bicubic) were implemented. In this work, the process of bilinear decimation is implemented. The phase of the element in the *m*-th line and *n*-th column, of the matrix *A_k_*, is the result of the average of a rectangular window of four pixels, this small window was chosen to have a minimum information loss at each decimation step. The following formula describes the operation:

(8) $$A_{k}\left(m,n\right)=\{\begin{array}{c} 1,\;if\;\alpha_{m,n}\;\geq 0.5,\;\\ 0,\;otherwise, \end{array}$$

where,

(9) $$\alpha_{m,n}=\frac{1}{4}\sum_{i=0}^{1}\sum_{j=0}^{1}A_{k-1}\left(2m+i,2m+j\right)$$

The analysis of the statistical response as a function of the decimation process [13], allowed determining an “optimal decimation step”, which is based on the correlation length ($l_{\beta ,j}$). $l_{\beta ,j}$ is defined as the range over which the descriptor *β* of the phase *j* approaches, to a certain extent, at the horizontal axis $\langle F_{\beta ,j,0}\rangle =0$ for the first time. In order to provide a good statistical representation, the gradual decimation process should be stopped at the optimal decimation step *Z*, given by Equation (10).

(10) $$Z=\left[min\left\{log_{2}\left(\frac{M_{0}}{M_{z}}\right),log_{2}\left(\frac{N_{0}}{N_{z}}\right)\right\}\right]$$

where,

(11) $$M_{z}=\left[\frac{3M_{0}}{l_{\beta ,j}}\right],\;N_{z}=\left[\frac{3N_{0}}{l_{\beta ,j}}\right]$$

For the experiments in this study, *k = 4* to compare the results on the descriptors and the ETC. The value of *Z* is determined in $k_{0}$, for further analysis.

### 2.3. Effective Transport Coefficient

The normalization and generalization of the results was done through the calculation of the resistivities to estimate a conduction efficiency ($\varepsilon_{k}$).

(12) $$\varepsilon_{k}=\frac{\Gamma_{eff}}{\Gamma_{M}}$$

As conductivity is the inverse of resistivity, the CL’s effective conductivity is the inverse of the effective resistivity value $\Gamma_{eff}=\frac{1}{\rho_{eff}}$. $\varepsilon_{k}$ is calculated by comparing the effective conductivity ($\Gamma_{eff}$) with the nominal conductivity ($\Gamma_{M}$), as described by Equation (12).

Although in this work only the ohmic conduction efficiency will be determined, other transport properties, such as thermal conductivity and diffusion coefficient, may be determined under the same approach.

## 3. Results and Discussion

Results are presented, according to the methodology, using four materials. First (S70) and second (S50) synthetic images are generated from random mathematical descriptors with its surface fraction controlled. Third (SEM1) and fourth (SEM2) images were obtained from SEM. In the case of the SEM image, they are preprocessed to obtain a binary representation using SVM (Support Vector Machine) [4].

Each 2D material is reconstructed to obtain a ω realization of a *Ω* ensemble of ten different random series (*W = 10*). Table 1 shows the main characteristics of microstructures for each material, image size and surface fraction.

### 3.1. Image Processing

**Figure 2:**
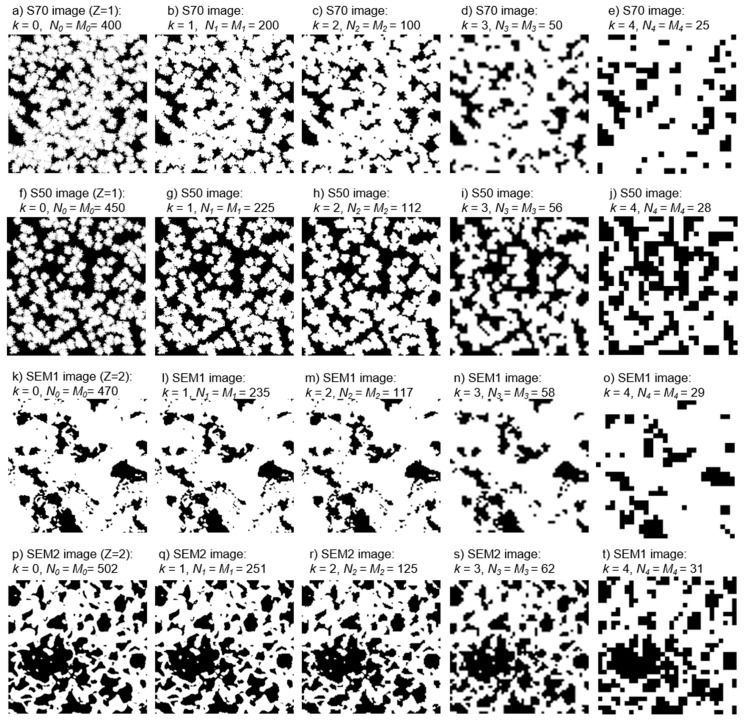
Decimation process of SEM and synthesized images for different *k* respect to full resolution image for each material.

The decimation process was applied using Equations (8) and (9). Figure 2 shows the original and processed images of different materials. The transformation, due to *k*-th decimation iteration, is observed along the columns. Despite the loss of detail with the decrease of the image, it is possible to identify the resemblance with respect to the original image. 

To preserve a statistical representation from the full resolution $F_{\beta ,j,0}$, the decimation level *k* should be less or equal to *Z* (Equation (10)) [13]. The *Z* value for each material is *Z = 1* for S70, *Z = 1* for S50, *Z = 2* for SEM1, and *Z = 2* for SEM2. 

### 3.2. Reconstruction and Statistical Analysis of Microstructures

**Figure 3:**
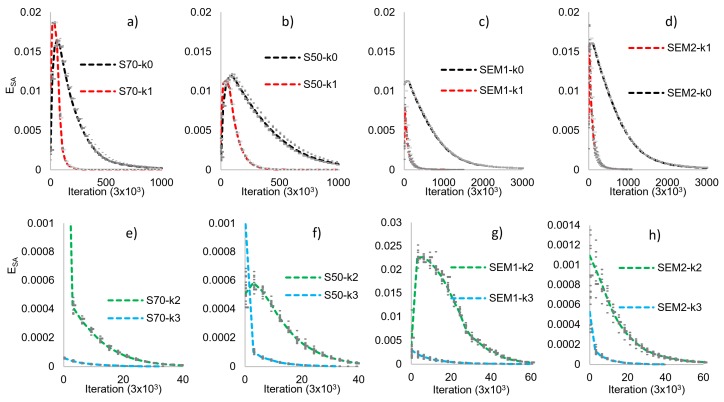
SA error (or energy) for each ω realization, *k*-th decimation, and ensembles for the studied materials. (**a**–**d**): k0 and k1 decimation and (**e**–**h**): k2 and k3 decimation.

Each microstructure is statistically characterized, in both phases, by $S_{j}$,$\;L_{j}$ and $P_{j}$ functions. Nevertheless, only $S_{j}$ and $L_{j}$ are applied to generate a *Ω* ensemble by SA reconstruction. Figure 3 shows the evolution of the E_SA_ determined at each 3 × 10^3^ iterations on the SA reconstruction process for all ω realizations. The “grey points” are plotted for all *ω* realizations and the dotted lines indicate the average error of reconstruction of the *k*-th decimation. The effect of the decimation on the number of iterations required for the convergence of reconstruction is clearly observed. In this work, error reconstruction convergence is 1 × 10^−6^. The decreasing value of iterations is directly associated with the image size reduction. In Figure 3, it is observed the nature of the reconstruction process, although the difference of the materials and the randomness of the *ω* realization, the individual errors are very closed of the average error. 

As the decimation process increases (increment in the value of *k*), the time is considerably reduced. A discrepancy in form, with respect to the trend displayed by the full-size image, is produced due to the lower number of pixels (values of the solution affected by the decimation process), which produces an abrupt fall with respect to the full-size image.

**Figure 4:**
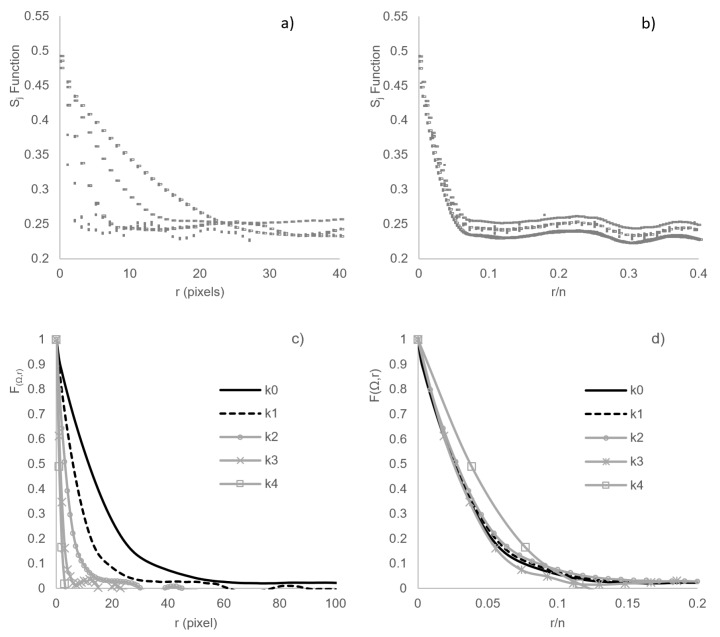
$S_{j}$ correlation function and $F_{\left(\Omega ,r\right)}$ average for all *ω* realizations of every configuration of the S50 image.

In order to show the behavior of the statistical characterization according to the decimation process, Figure 4 shows the individual and average response as a function of *r* and *r/n*. Also, in Figure 4, it is observed how the SA reconstruction is implemented in conjunction with the decimated process. With the aim to simplify the analysis, only S50 material is presented as a representative sample, since the other materials have the same behavior. 

Figure 4a shows the $S_{j}$ function which is plotted with respect to *r* and Figure 4b shows $S_{j}$ function with respect to *r/n*, for all *ω* realizations of the four decimations (*k0–k4*). In Figure 4a, it is observed that the value of the surface fraction ($\emptyset_{j}=S_{j}\left(r=0\right)$) does not have a representative change with the decimation. The average value, for all *ω* configurations for all decimation steps (*k0–k4*), is $\emptyset_{j}=0.49$, with a variance of 1.3%. In Figure 4a,b, similar to Figure 3, the convergence term used in the SA reconstruction is (1 × 10^−6^), allowing the statistical reproduction of the system. In this regard, two aspects are indicated: (1) in both images 4a and 4b, the variation between *ω* configurations of the same *Ω* ensemble does not significantly affect the response of the $S_{j}$ function. (2) Figure 4b shows the trend of the behavior of the *Sj(r/n)* function, regardless of the decimation stage.

Figure 4c,d show the global average ($F_{\left(\Omega ,r\right)}$). In Figure 4c, $F_{\left(\Omega ,r\right)}$ is plotted with respect to *r* and, in Figure 4d, it is plotted with respect to *r/n*. The behavior of Figure 4a,c are similar, responding in a consistent manner to what is expected in a decimation process, where the image size is reduced, the length of the $F_{\left(\Omega ,r\right)}$ is also reduced. It is important to mention that this distance is affected by decimation, but it is not the characteristic length ($l_{\beta ,j}$) used in Equation (11) [13]. In Figure 4d, a slight separation is observed between the response of the original image *k0* with respect to the decimated images. According to previous work [13], the response of normalization coarseness (Equation (7)) allows making the quantitative proposal of the loss of information based on the decimated process and the application of the methodology to determine *Z* (Equations (10) and (11)).

**Figure 5:**
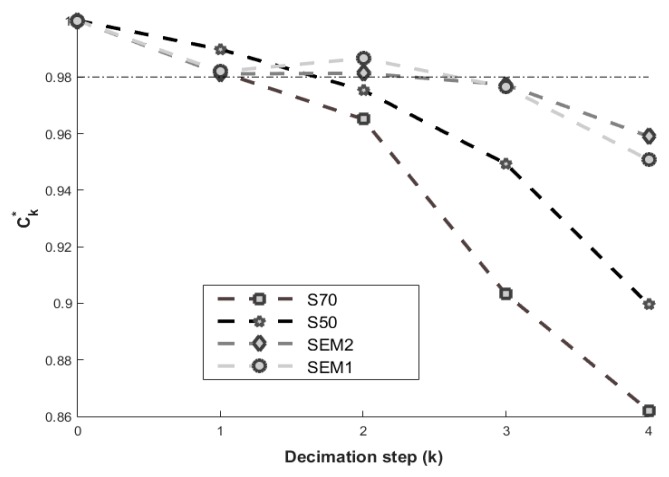
Normalized Coarseness for the first 5 values of the decimation process (*k*) applied to S70, S50, SEM1, and SEM2 figures.

When *Z* is considered as the maximal *k* value ($k_{max}$), statistical descriptors do not suffer big effects, independently of the particular characteristics of each material (distribution of the pixels, surface fraction, as well as the decimation method) [13]. In Figure 5, the normalization coarseness (Equation (7)), for different *k* values, of the four samples is presented. The indicated region, with a reference line at 0.98, denotes the loss of information that does not have a great impact on the results (2%). 

The lower value of $C_{k}^{*}$ means that more information was lost. In the dotted line of Figure 5, it is observed that k=1 reaches a value between 1.00 and 0.98, for the four materials. However, they have a value between 1.00 and 0.98 for SEM1 and SEM2, while for materials S50 and S70 they have a value of less than 0.98. This coarseness value is linked to the computation of *Z*, and it indicates that decimation k=2 of images S50 and S70 will lose an unacceptable amount of statistical information.

### 3.3. Effective Transport Coefficient

**Figure 6:**
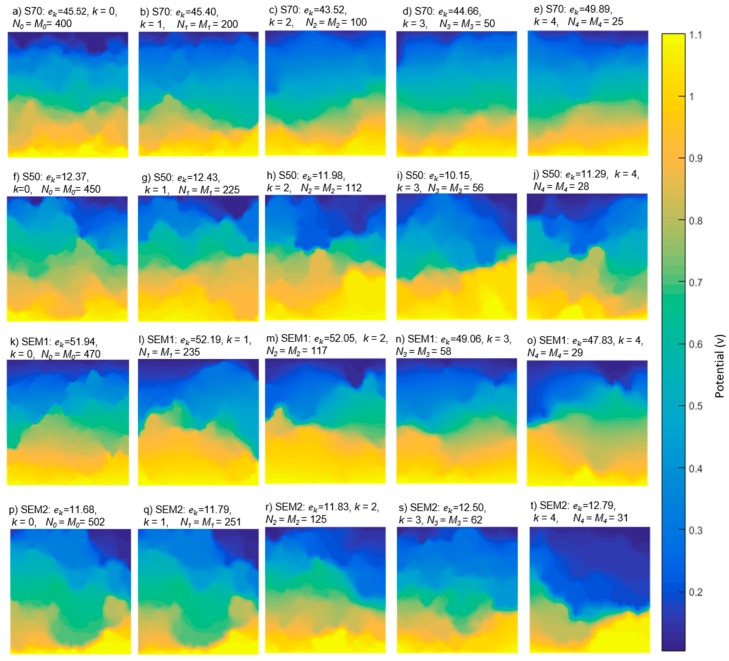
Potential distribution (V) of the electronic conduction phase of some reconstructed samples. (**a**–**e**): S70 image, (**f**–**j**): S50 image, (**k**–**o**): SEM1 image and (**p**–**t**): SEM2 image.

The method used to compute $\varepsilon_{k}$ is obtained through FVM [1] and Equation (12). The initial conditions of the method are the parameters of the reconstructed microstructures, which are introduced in the computation region in order to obtain their ETC and, subsequently, their $\varepsilon_{k}$. Ten *ω* realizations were considered for each *Ω* ensemble for a total of 200 reconstructions. Figure 6 shows a representative solution of the distribution of the potential, for the phase j=0 of the four materials, and their respective decimated samples with respect to the parameters described in Figure 2.

**Figure 7:**
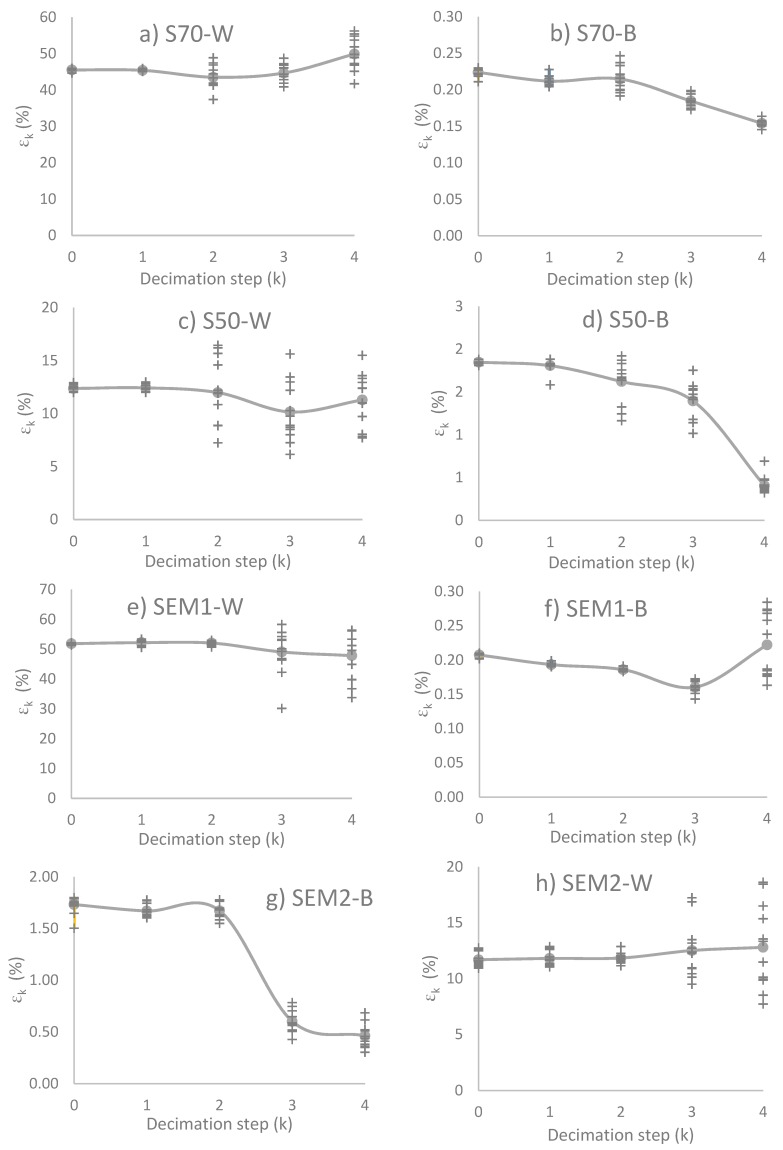
Conduction Efficiency ($\varepsilon_{k}$). (**a**): White phase of S70 image, (**b**): Black phase of S70 image, (**c**): White phase of S50 image, (**d**): Black phase of S50 image, (**e**) White phase of SEM1 image, (**f**): Black phase of SEM1 image, (**g**): White phase of SEM2 image, (**h**): Black phase of SEM2 image.

In Figure 6, each image corresponds to a representative microstructure, but $\varepsilon_{k}$ value is the average of the ten *ω* realizations, which is identical in Figure 7. Figure 7 shows the $\varepsilon_{k}$ evolution on both phases as a result of the decimation process, the “points” plotted correspond to all *ω* realizations of the structures reconstructed and the lines indicate the average on the *Ω* ensemble. The standard deviation, in phase j=1, increases when the decimation value increases, causing a dispersion for decimate value greater than $Z=k_{max}$. The phase j=0 presents less conduction, diminishing in ETC values. However, in the case of j=0, the dispersion phenomenon is less for high *k* values.

The change in image resolution, due to intentional decimation, modifies the average $\varepsilon_{k}$ value and the standard deviation value. For each material, when k=1, the $\varepsilon_{k}$ average is statistically equal to k=0 with a low standard deviation. Likewise, when k≥3, the average $\varepsilon_{k}$ values change, the standard deviation is high. When analyzing the decimated k=2, materials S50 and S70 yield a statistically different $\varepsilon_{k}$ value than the original image and have a high standard deviation. While the SEM1 and SEM2 materials, decimated at k=2, have a statistically similar value to the original image and have a low standard deviation. These $\varepsilon_{k}$ results are related to the values of normalized coarseness, but more importantly, its behavior is related to the maximum decimation value that preserves the statistical descriptors.

## 4. Conclusions

This paper presents a new methodology for RHM reconstruction to obtain the conduction efficiency in two-phase materials. SEM or synthetically generated images are the sources to perform such reconstruction. Image size is reduced by a factor of 2, with an iterative decimation process. At each step, a comparison between the contained information of the respective image and the original one using the normalized coarseness is made. The optimal number of decimation steps is selected as the biggest number that has a normalized coarseness above 0.98, which allows obtaining a reconstruction statistically equivalent to the original images and with the same (statistical) conduction efficiency. Statistical descriptors are computed for the original image, as well as for images that have been decimated. Depending on the analyzed image, its maximum decimation value ($Z=k_{max}$) is obtained to calculate the randomly reconstructed structures and then simulate, through of Finite Volume Method, the conduction efficiency.

The morphological information obtained is used to create study limits for a variety of effective properties of random media. This information is mainly constituted by the distribution of pore size along the surface of the material, leading to the importance of understanding the fundamental relationship between a correlation function for an experimental material and a synthetic one, because the synthetic and experimental data were digitized with a finite resolution.

One of the advantages of using this method is that both simulation times and memory usage decrease when performing decimation on an image. By decreasing computational resources, the size of the simulations can be scaled.

Finally, results show the evolution of the reconstruction method including a decimation process providing a very close approximation with the statistical descriptors obtained from the correlation functions of the structures and its relationship with the progress of conduction efficiency. If the maximum decimation ($Z=k_{max}$) is respected for each material, the statistical behavior and conduction efficiency will be within the appropriate parameters. 

## Figures and Tables

**Table 1:** Characteristics of the four images used.

Name	Size (Pixel)	$\mathrm{Surface}\;\mathrm{Fraction}\;\phi_{j}\;(\%)$
S70	400 × 400	70
S50	450 × 450	50
SEM1	470 × 470	78.20
SEM2	502 × 502	80.48
